# Incidence of type 1 and type 2 diabetes before and during the COVID-19 pandemic in Germany: analysis of routine data from 2015 to 2021

**DOI:** 10.25646/11730

**Published:** 2023-11-08

**Authors:** Lukas Reitzle, Christin Heidemann, Josephine Jacob, Dorota Pawlowska-Phelan, Marion Ludwig, Christa Scheidt-Nave

**Affiliations:** 1 Robert Koch Institute, Berlin, Germany Department of Epidemiology and Health Monitoring; 2 InGef – Institute for Applied Health Research Berlin GmbH, Germany

**Keywords:** TYPE 1 DIABETES, TYPE 2 DIABETES, INCIDENCE, TREND, COVID-19, GISD, DIABETES SURVEILLANCE

## Abstract

**Background:**

To date, there is no data available depicting the trend of the incidence of type 1 and type 2 diabetes across all age groups for the COVID-19 pandemic years in Germany.

**Methods:**

Based on anonymized routine data from nine million persons covered by statutory health insurance, newly diagnosed diabetes cases (ICD diagnosis E10.- to E14.-) in inpatient or (confirmed in two quarters) outpatient setting were estimated for 2015 to 2021, differentiating between type 1 and type 2 diabetes. The data were linked to the German Index of Socioeconomic Deprivation. The results are age-standardised (population as of 31 Dec. 2021).

**Results:**

Between 2015 and 2021, the incidence of type 1 diabetes increased from 9.5 to 11.6 per 100,000 persons (from 7,007 to 8,699 new cases per year). In contrast, the incidence of type 2 diabetes tended to decline between 2015 and 2019. It continued to drop initially in 2020 during the pandemic, and then rose to 740 per 100,000 persons in 2021 (556,318 new cases per year). The diabetes type-specific seasonal pattern of previous years has changed during the pandemic years. The incidence of both type 1 and type 2 diabetes was observed to be higher in regions of high socioeconomic deprivation as compared to regions characterised by low socioeconomic deprivation.

**Conclusions:**

The increase in the incidence of type 1 and type 2 diabetes in 2021 may possibly be related to the COVID-19 pandemic. The high incidence and the differences by regional socioeconomic deprivation indicate that there is a need for targeted prevention strategies.

## 1. Introduction

Diabetes mellitus is a group of metabolic diseases that have a reduced effect or an impaired production of the hormone insulin as a common characteristic, which leads to elevated blood glucose levels. Type 1 and type 2 diabetes are the main forms of diabetes. Rare forms include genetic or drug-induced diabetes and gestational diabetes, which usually resolves after pregnancy but increases the risk of developing type 2 diabetes later in life [1].

Type 1 diabetes is usually based on an autoimmune disease that often develops already in childhood and adolescence. Autoantibodies destroy the insulin-producing cells of the pancreas resulting in little or no insulin production by the body itself (absolute insulin deficiency). This means that the external supply of insulin is vital for treatment of type 1 diabetes [2]. The most common type of diabetes, type 2 diabetes, usually develops in middle or old age due to the interplay of various risk factors. These include a lifestyle characterised by unfavourable eating habits and physical inactivity, obesity as well as genetic factors. The effect of insulin is reduced in type 2 diabetes (insulin resistance), so that more insulin must be produced (relative insulin deficiency) to keep blood glucose level constant. The treatment of type 2 diabetes involves lifestyle changes or drug therapy, depending on the specific risk factors and metabolic profiles [3].

Both type 1 and type 2 diabetes can be associated with severe acute metabolic complications. This can be compounded by long-term complications, particularly kidney disease (nephropathy), eye disease (retinopathy), nerve disease (neuropathy) and cardiovascular disease. These complications can contribute significantly to a reduced quality of life and shorter life expectancy of those afflicted, as well as extensive costs incurred for the healthcare system [4, 5].

Estimates from the International Diabetes Federation’s Diabetes Atlas 2021 indicate that a total of 537 million people aged 20 to 79 years are affected by diabetes globally. This corresponds to 10.5 % of the world population in this age range [6]. According to calculations of the Global Burden of Disease (GBD) study almost 23 million new diabetes cases were reported in 2017. This is equivalent to 285 new cases per 100,000 persons [7]. While just under 2 % of the new cases are type 1 diabetes, type 2 diabetes accounts for the vast majority of new cases [7].

The proportions of existing cases (prevalence) and new cases (incidence) of diabetes in the population are key indicators of the diabetes surveillance. Close surveillance over time is of great importance for adequate planning of prevention and care measures. Investigating trends over time, known differences between different population groups, especially differentiated by age, sociospatial and geographical region, must be taken into account [6–8]. But influencing factors such as demographic changes, adjustments to the criteria for diabetes diagnosis, or crises, such as the recent COVID-19 pandemic, also play a role. However, for most countries current trend analyses of the incidence of type 1 and type 2 diabetes over the time of the COVID-19 pandemic and for all age groups of the population are not yet available.

Against this background, the aim of the present study was to map the development of diabetes incidence over time in Germany as part of a collaborative project of the diabetes surveillance at the Robert Koch Institute [9]. The incidence of diabetes was estimated on the basis of anonymized routine data of persons covered by statutory health insurance, differentiated by type 1 or type 2 diabetes for the years 2015 to 2021, both overall and differentiated by sex and age groups as well as by sociospatial differences. In order to better understand any possible influence of the COVID-19 pandemic on the current development of the incidence, a quarterly view of the incidence of type 1 and type 2 diabetes is provided in addition to the annual view.

## 2. Methods

### 2.1 Data basis

The present analyses were based on the research database (formerly HRI database) of the Institute for Applied Health Research Berlin GmbH (InGef), which contains anonymized routine data for approximately nine million people covered by statutory health insurance in Germany (data from approximately 57 guild- and company-based health insurance funds) [10]. In addition to sociodemographic data on age, sex and place of residence, the data set contains information on outpatient and inpatient diagnoses and services, on occupational disability as well as on prescriptions for medicines and remedies and on costs incurred in the respective settings. Diagnoses and medications are coded according to the International Statistical Classification of Diseases and Related Health Problems (ICD-10) and the Anatomical Therapeutic Chemical (ATC) Classification System, respectively. The analyses of the years 2019 to 2021 investigating regional socioeconomic deprivation were based on the German Index of Socioeconomic Deprivation (GISD) Release 2022 v0.1 [11, 12]. The GISD was linked to the place of residence of the health-insured persons at the district level using the Official Municipal Code.

### 2.2 Study design and study population

For the present study, persons with health insurance coverage between 2015 to 2021 were considered cross-sectionally for each reporting year. Insured persons who were fully observable throughout the respective year of the study or from birth until 31 Dec. of the study year were included. Furthermore, information on the calendar year prior to the study year (pre-observation period) had to be available for all included individuals, either from birth to 31 Dec. or for the entire year. Persons with a diagnosis of diabetes (ICD: E10.- to E14.-) in an outpatient or inpatient setting in the pre-observation period (four quarters before the respective study year) were excluded from the analysis. Only confirmed diagnoses were taken into account in the outpatient setting, and both main and secondary diagnoses in the inpatient setting. Furthermore, persons who had a prescription for medication for treatment of diabetes (anti-diabetics: ATC codes A10A or A10B) in the pre-observation period were excluded.

### 2.3 Definition of diabetes incidence and differentiation between types

Incident diabetes was defined as a documented E10.- to E14.- ICD diagnosis as the main diagnosis in the inpatient setting or as a confirmed diagnosis in one quarter of the study year in the outpatient setting, confirmed by another outpatient or inpatient diagnosis within the three subsequent quarters (m2Q criterion). An inpatient secondary diagnosis was considered to be equivalent to a confirmed outpatient diagnosis. Subsequently, the cases were classified into type 1, type 2 or other diabetes according to the algorithm for differentiation of diabetes types in routine data [13]. This is based on the documentation of outpatient and inpatient diabetes diagnoses, the prescription of anti-diabetic drugs, differentiating between insulin (ATC code: A10A) or other anti-diabetic drugs (ATC code: A10B), as well as age and the specific diagnostic pattern. The detailed assignment algorithm is described in Table 1 in the Annex. In addition, a distinction was made within the type 2 diabetes group between persons with at least one prescription of an anti-diabetic drug (ATC code: A10) within the first year of treatment and persons without medication.

### 2.4 Statistical analyses

The incidence was estimated for each reporting year as the number of persons with newly diagnosed diabetes relative to all persons at risk in the respective year, and the 95 % confidence intervals were calculated assuming a binomial distribution. The incidence in a reporting year was reported stratified by diabetes type (type 1, type 2), age group, sex and quarter in which the initial diabetes diagnosis was made. If diagnoses were made in several quarters, the time of the first documented diagnosis was considered to be the time of diagnosis. In the presentation of data by age groups, the groups < 7 years, 7 to 10 years, 11 to 13 years, 14 to 17 years, 18 to 34 years, 35 to 49 years, and ≥ 50 years were distinguished for type 1 diabetes, and the groups < 18 years, 18 to 34 years, 35 to 49 years, 50 to 64 years, 65 to 79 years, and ≥ 80 years were considered for type 2 diabetes. As the number of cases was low, the age groups for type 1 diabetes were combined into < 18 years and ≥ 18 years and for type 2 diabetes into < 35 years, 35 – 49 years, 50 – 64 years, and ≥ 65 years. The incidence in the 2019 to 2021 reporting years was also evaluated by regional socioeconomic deprivation of residence (categories: low = 20 % of districts with the lowest deprivation, medium = 60 % of districts with medium deprivation, high = 20 % of districts with the highest deprivation). To compensate for differences in age and sex distribution compared to the general population, the incidence was extrapolated using the German population as of 31 Dec. of the respective reporting year based on data of the Federal Statistical Office (Destatis) as reference population [14]. In addition, the incidence was age-standardised using the population as of 31 Dec. 2021. For extrapolation and standardisation, a population subdivision by 1-year age groups was applied, the weights of which were subsequently added up in the respective age groups. Microsoft R Open, version 4.0.2 was used as the statistical software for the evaluation.

## 3. Results

### 3.1 Study population

**Figure 1:**
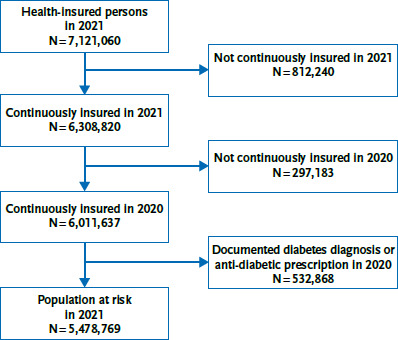
Flowchart for analysis of the incidence of type 1 and type 2 diabetes exemplary for the year 2021 Source: own illustration

After excluding all health-insured persons who were not observable during the observation period or in the previous year or who already had a previous diabetes diagnosis made, 5.4 to 5.7 million persons were included in each reporting year (Figure 1 and Annex Table 2). On average, extrapolated to the population of the respective reporting year, a diabetes was newly documented for 0.7 % of the included persons. The majority of cases were type 2 diabetes (96.2 %). Some 1.5 % were classified as type 1 diabetes and 2.3 % as other forms of diabetes.

### 3.2 Incidence of type 1 diabetes

**Figure 2:**
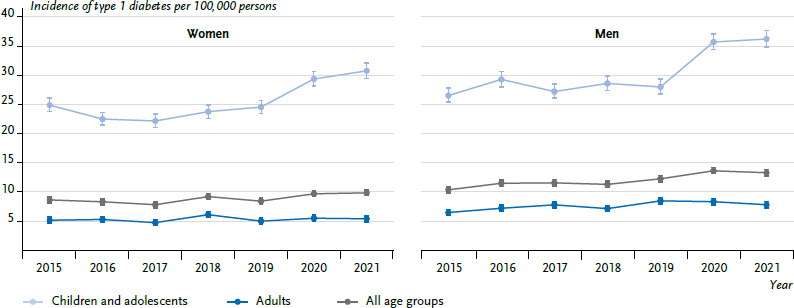
Temporal trend of the age-standardised incidence of type 1 diabetes per 100,000 persons with 95 % confidence interval by sex and age group (n per year: Annex Table 3) Source: InGef Research Database

The age-standardised incidence of type 1 diabetes increased from 9.5 (women: 8.7; men: 10.4) new cases per 100,000 persons in 2015 to 11.6 (women: 9.9; men: 13.3) new cases per 100,000 persons in 2021 (Figure 2). This corresponded to 7,007 persons in 2015 (women: 3,228; men: 3,779) and 8,699 persons in 2021 (women: 3,790; men: 4,908) newly diagnosed with type 1 diabetes. Both the absolute and the relative incidence of type 1 diabetes were higher in men than in women. The age-differentiated presentation shows that the increase was mainly due to the younger age groups. For example, the incidence of type 1 diabetes in children and adolescents (< 18 years) rose from 25.8 new cases per 100,000 persons in 2015 (girls: 24.9; boys: 26.5) to 33.6 new cases per 100,000 persons in 2021 (girls: 30.8; boys: 36.2). In adults (≥ 18 years), though, the increase in the incidence from 5.8 new cases per 100,000 persons in 2015 (women: 5.2; men: 6.5) to 6.6 new cases per 100,000 persons in 2021 (women: 5.4; men: 7.8) was less pronounced (Figure 2 and Annex Table 4 with more detailed differentiation of age groups).

**Figure 3:**
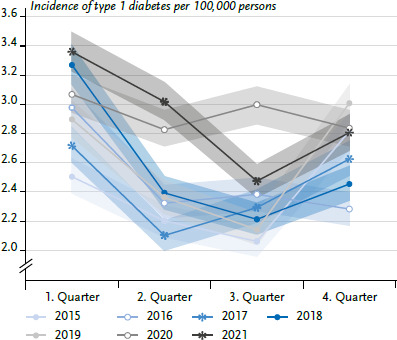
Age-standardised incidence of type 1 diabetes per 100,000 persons with 95 % confidence interval by quarter and reporting year (n per year: Annex Table 3) Source: InGef Research Database

Looking at the incidence of type 1 diabetes in the individual quarters of each year, a typical pattern emerged: at its highest in the first quarter, the incidence then dropped off in the second and third quarters, before it rose again in the fourth (Figure 3). In the 2020 and 2021 reporting years this seasonal pattern was changed, though. The type 1 incidence was higher than expected in the second and third quarters of 2020 and also in in the second quarter of 2021.

Stratified by regional socioeconomic deprivation, the type 1 diabetes incidence is higher in regions with high socioeconomic deprivation than in regions with medium or low deprivation in all reporting years (Annex Table 5). In 2021, the incidence of type 1 diabetes per 100,000 persons was 15.9 (women: 13.1; men: 18.7) in regions with high deprivation compared to 11.0 (women: 9.2; men: 12.7) in regions with medium and 11.1 (women: 10.2; men: 12.2) in regions with low deprivation. The differences between regions of low and medium deprivation are small and tend to be inconsistent over the reporting years. Differentiated by age, the regional socioeconomic differences in the incidence of type 1 diabetes are most pronounced in childhood and adolescence and less so in adulthood (Annex Table 5).

### 3.3 Incidence of type 2 diabetes

In 2015, the age-standardised incidence of type 2 diabetes was 0.74 % (women: 0.68 %; men: 0.80 %), dropping in subsequent years to 0.67 % (women: 0.61 %; men: 0.74 %) in 2017, and then remaining steady until 2019. The incidence of type 2 diabetes declined in the first year of the COVID-19 pandemic and then rose significantly to 0.74 % in 2021 (women: 0.67 %; men: 0.81 %). In 2021, this corresponded to 556,318 persons (women: 257,486; men: 298,832) with a newly documented type 2 diabetes. Men are diagnosed with type 2 diabetes more frequently than women. Differentiating by age groups, the decline in the incidence of type 2 diabetes from 2015 to 2017 was most pronounced in the age groups above 50 years, while the incidence was fairly constant in the younger age groups (Figure 4A and Annex Table 4 with a more detailed differentiation of age groups). Likewise, the strong decline in the incidence in 2020 and the subsequent increase was noted mainly in the older age groups.

A further analysis considered only those persons with type 2 diabetes, who were treated with medication in the quarter of diagnosis or the three subsequent quarters. Unlike the overall incidence of type 2 diabetes, the incidence of type 2 diabetes treated with medication remained relatively constant between 2015 and 2020, increased in the age groups under 50 years and dropped in the older age groups (Figure 4B). In 2021, the incidence of type 2 diabetes treated with medication increased significantly in all age groups.

**Figure 5:**
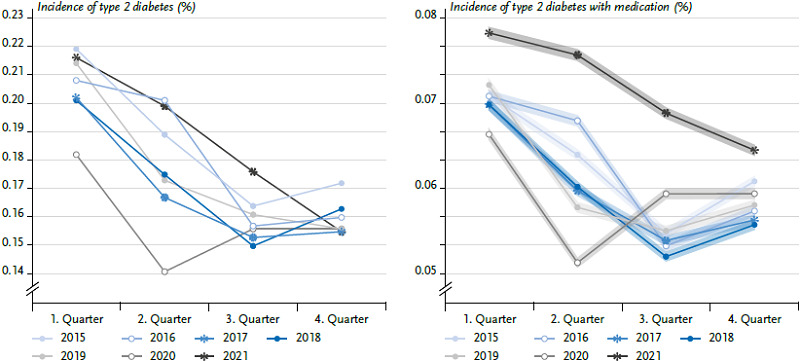
Age-standardised incidence of type 2 diabetes overall (left) and treated with medication (right) in % with 95 % confidence interval by quarter and reporting year (n per year: Annex Table 3) Source: InGef Research Database

A specific seasonal pattern also emerged for the incidence of type 2 diabetes in the analysis of the individual quarters of the reporting years. The incidence of type 2 diabetes was highest in the first quarter, dropped in the second and reached a plateau in the third and fourth quarters (Figure 5). The same seasonal pattern is not seen in 2020 and 2021: in 2020, the incidence dropped more sharply in the second quarter than in previous years, before it rose again in the third quarter. In 2021 the incidence fell more slowly than in previous years and failed to reach a plateau in the third and fourth quarters, though. For type 2 diabetes treated with medication, the seasonal pattern was similar, but more pronounced (Figure 5). Here, the incidence of type 2 diabetes treated with medication in 2021 was higher than in previous years in all quarters.

There also were clear differences in the incidence of type 2 diabetes stratified by socioeconomic deprivation (Annex Table 5). In 2021, the incidence of type 2 diabetes was 0.67 % in regions with low socioeconomic deprivation (women: 0.61 %; men: 0.73 %), increasing with more pronounced deprivation to 0.87 % (women: 0.82 %; men: 0.93 %) in regions with high deprivation. This difference is evident in all reporting years presently analysed.

## 4. Discussion

The average incidence of (all types of) diabetes based on anonymized routine data from about nine million persons covered by statutory health insurance was 710 new cases per 100,000 persons (0.7 %) per year in Germany for the period of 2015 to 2021. Type 2 diabetes accounted for the majority of new cases (96.2 %). Type 1 diabetes and other forms of diabetes accounted for 1.5 % and 2.3 % of new cases, respectively [7].

The present results demonstrate an increase in the incidence of type 1 diabetes from 9.5 to 11.6 per 100,000 persons between 2015 and 2021. This corresponds to approximately 8,700 persons newly diagnosed with type 1 diabetes in Germany in 2021. In the context of the COVID-19 pandemic, the incidence of type 1 diabetes increased beyond expectations in 2020. The incidence of type 2 diabetes initially declined between 2015 and 2017 and then remained at the same level until 2019. During the pandemic year 2020, the incidence dropped to 629 per 100,000 persons (0.6 %), followed by a considerable increase to 740 per 100,000 persons (0.7 %) in 2021. This corresponds to about 560,000 new cases of type 2 diabetes in 2021.

### 4.1 Interpretation of the results on type 1 diabetes

#### Differences by age, sex and regional socioeconomic deprivation

On a global level, 400,000 new cases of type 1 diabetes (5 new cases per 100,000 persons) were estimated for 2017 based on the Global Burden of Disease (GBD) study. The incidence was subject to large regional variation [7], which is consistent with other studies [15]. Germany was one of the regions with the highest incidence of type 1 diabetes. For 2019, the GBD study reported an incidence of type 1 diabetes of 11.3 per 100,000 persons (equivalent to 9,599 new cases) in Germany [16], which is slightly more than in the present analysis (10.3 per 100,000 persons).

Consistent with the results of the present study, the incidence of type 1 diabetes per 100,000 persons in the GBD study was higher in childhood and adolescence (< 20 years: global: 11.2; Germany: 24.5) than in adulthood (≥ 20 years: global: 5.5; Germany: 8.3) and it was higher in men (global: 7.8; Germany: 12.0) than in women (global: 6.9; Germany: 10.7). A review of data from 92 countries also shows that the incidence of type 1 diabetes in childhood and adolescence was higher in boys than in girls and was highest among 5- to 14-year-olds [15]. The sex-specific difference in the incidence is discussed in association with differences in genetic predisposition, hormonal factors and sensitivity to environmental factors that may promote type 1 diabetes [15]. Current estimates for Germany based on the nationwide Diabetes Prospective Follow-up (DPV) registry and regional diabetes registries indicate an incidence in childhood and adolescence (0 to 17 years) of 26.5 for girls and 31.9 for boys per 100,000 persons in 2020, with the incidence being highest in the age group of 7 to 13 years [17]. The data is scarce for the incidence of type 1 diabetes in adulthood for international comparison, especially for countries with low to middle range incomes. A study based on incidence data of type 1 diabetes in adults (≥ 20 years) from 32 countries and regions for the period from 1973 to 2019 showed no unambiguous decrease in the incidence with increasing age, but a higher incidence in men as compared to women [18]. For Germany, registry data for the adult age range indicate a higher incidence of 5.8 for men as compared to 5.4 for women per 100,000 persons for 2014 to 2016, whereby the incidence steadily declined with increasing age [19]. Comparing the corresponding years and age groups, the results of the present study are very similar.

In addition to regional differences throughout the world, differences according to country-specific income levels were reported, with higher incidence estimates for higher-income countries than for lower-income countries [15]. Within individual countries, differences according to population density were noted, with incidence being lower in more densely populated areas than in sparsely populated areas, though this was observed only in some studies, but not in others [20]. Using the German Index of Socioeconomic Deprivation (GISD), a higher incidence in regions with high deprivation than in those with low deprivation was found for children and adolescents based on registry data in Germany, which is consistent with our study [17]. However, a regional study from North Rhine-Westphalia observed no association between the German Index of Multiple Deprivation (which takes into account other domains of deprivation in addition to socioeconomic deprivation, e.g. on safety and the environment) and the incidence of type 1 diabetes, and even demonstrated an inverse association with the incidence for the environmental domain [21]. Overall, these results support the hypothesis that environmental factors play a role in the development of type 1 diabetes, with different factors being discussed to date (especially factors acting in the foetal, infant and early childhood phases, such as viruses and other pathogens, certain nutrients and breastfeeding, pollutants and climatic factors). However, as the data is inconsistent, further research is needed to elucidate the underlying mechanisms [20] and to be able to better interpret the difference in results related to different indices of deprivation.

It is worth noting that despite the much higher incidence per 100,000 persons in childhood, a large number of new cases still occur in adulthood as this fraction of the population is larger: 58 % of all new cases worldwide concern people aged 15 years and older [8]. Interpreting comparisons between countries, it should also be noted that the proportion of undiagnosed new cases of type 1 diabetes varies greatly. This proportion is estimated to be very high in countries with low income levels, whereas it is only about 5 % of all new cases of type 1 diabetes in Western Europe [22].

#### Temporal trend with a view to the COVID-19 pandemic

Reviews of trends in the incidence of type 1 diabetes between the mid-1980s and the mid-2010s in children (< 15 years of age) mainly indicate that there was an increase, which was 3 to 4 % on average per year worldwide. This was most pronounced in the group of 0- to 4-year-old children [24]. In contrast, the data on trends in adults is insufficient and the few studies available do not indicate a consistent trend [18]. But these reviews on long-term trends do not yet include the two past years of the COVID-19 pandemic, i.e. 2020 and 2021.

In terms of the pandemic years, individual studies indicate that the incidence of type 1 diabetes was higher than expected based on the earlier trend [25–28]. For example, a German study found the incidence in children and adolescents (< 20 years), defined as first-time insulin prescriptions, to be 44 % and 65 % higher than expected in the summers of 2020 and 2021, respectively, resulting in less seasonal variation in the type 1 diabetes incidence [25]. The present study also shows an increase in the incidence of type 1 diabetes in 2020 and 2021, most pronounced in children and adolescents. Looking at the quarters, in which the diagnosis was made, there was no decrease in incidence at the onset of the pandemic in the second quarter of 2020. Also, the typical seasonal pattern, with lowest incidence usually in the second and third quarters, was not observed at all in 2020 and was less pronounced in 2021. Since no correlation between the incidence of COVID-19 and the incidence of type 1 diabetes was detected in the German study mentioned above, indirect effects of the COVID-19 pandemic were suspected as possible causes raising the type 1 diabetes incidence. Containment measures or fear of infection may have promoted stress, which in turn may have increased the risk of developing an autoimmune response [25]. In addition, due to the acute onset of symptoms upon manifestation of type 1 diabetes, a medical consultation is needed.

Furthermore, a direct association between a SARS-CoV-2 infection and the development of type 1 diabetes has not yet been conclusively confirmed. The results of current studies are inconsistent. For example, a meta-analysis of cohort studies showed that individuals diagnosed with COVID-19 had a higher risk of an initial diagnosis of type 1 diabetes compared to controls without COVID-19 [29]. An increased risk of initial diagnosis of an autoimmune disease, including type 1 diabetes, after a documented COVID-19 disease was noted in a German cohort study based on data from individuals covered by statutory health insurance [30]. However, a recent Danish cohort study of children and adolescents (< 18 years) based on nationwide registry data did not observe an increased risk of developing type 1 diabetes after SARS-CoV-2 infection [31]. Possible causes under discussion include inflammatory processes that promote insulin resistance and might also directly damage the pancreatic β-cells [29] as well as persistent autoantibodies and serological autoreactivity [30].

### 4.2 Interpretation of the results on type 2 diabetes

#### Differences by age, sex and regional socioeconomic deprivation

The GBD study estimated for 2017 a total of 22,535,000 new cases of type 2 diabetes across all age groups (279 new cases per 100,000 persons), again with large regional differences being evident [7]. Based on the GBD data for 2019, a comparatively high incidence is reported for Germany (509 new cases per 100,000 persons, 432,005 new cases in total) [16], which is, however, lower than the estimate from the present study (698 new cases per 100,000 persons). According to global estimates, there were more new cases of type 2 diabetes in men (11,549,000) than in women (10,987,000) [7]. The incidence of type 2 diabetes increased from the age group of 10 to 14 years to its highest value in the age range of 55 to 64 years and then decreased slightly [7]. Only scarce data is available on the incidence of type 2 diabetes in children and adolescents (< 20 years) and estimates vary considerably [16, 32].

The estimates available for Germany based on outpatient accounting data range from 450,000 to 540,000 new cases of type 2 diabetes each year [33, 34]. For 2019, the incidence was estimated to be 6.1 per 1,000 persons for women and 7.7 per 1,000 persons for men, which is in line with the present results [34]. Furthermore, both the present data and analyses of outpatient accounting data demonstrate an increase in incidence with increasing age reaching a peak between 70 and 80 years. Moreover, the incidence of type 2 diabetes was consistently higher in men than in women in the age groups of 40 years and older [33, 34]. This sex difference in the incidence of type 2 diabetes is probably related to differences in the profile of behavioural risk factors [35] and glucose and lipid metabolism [36]. Nationwide estimates for the type 2 diabetes incidence of children and adolescents in Germany based on registry data indicate that there were 2.8 new cases per 100,000 person-years (approximately 175 new cases annually) in the age group 11 to 18 years in 2014 to 2016 [19]. However, in the present study and in an analysis of outpatient accounting data, the incidence of type 2 diabetes in children and adolescents is substantially higher [33]. These deviations might be related to differences in the recording methodology and classification of type 2 diabetes.

In addition to the known regional differences in the incidence of type 2 diabetes on a global level [2], differences according to regional deprivation have reported within countries [37–39]. For example, a Scottish study showed that new cases of type 2 diabetes were more frequent in adults living in deprived areas as compared to non-deprived areas [37]. A Finnish study indicated that children from socioeconomically deprived neighbourhoods had less favourable lifestyle factors in their childhood and adolescent life compared to those from less deprived neighbourhoods. Persistent socioeconomic deprivation increased the risk of developing type 2 diabetes in middle adulthood [38]. For Germany, a higher modelled type 2 diabetes incidence was shown for high deprived regions as compared to low deprived regions by applying the German Index of Multiple Deprivation [40]. The rising incidence of type 2 diabetes with increasing regional socioeconomic deprivation as observed in our study is consistent with these results. This points to the importance of the living environment for the onset of health risk factors that contribute to the development of type 2 diabetes.

#### Temporal trend with a view to the COVID-19 pandemic

Reviews indicate that the incidence of type 2 diabetes has consistently increased in low- and middle-income countries between the 1990s and the 2010s [7]. This has been linked to demographic ageing and changes in dietary behaviour and physical activity [7]. In contrast, for most high-income countries, the incidence of type 2 diabetes first increased into the 2000s and then stagnated or dropped until 2017 [7, 41, 42]. Changes in diagnostic criteria, but also prevention measures and education campaigns may have contributed to the latter observation [41, 42]. Accordingly, a decrease in the lifetime risk of type 2 diabetes was observed in 11 out of 15 regions in Europe, Asia, North America and Australia with mostly high income levels and available data for 2005 to 2019 [43] and, similarly, in Germany the average 5-year risk of type 2 diabetes decreased between the late 1990s and the 2010 [35].

The data on the development over time of the incidence of type 2 diabetes in Germany to date is fragmented. Few available results suggest an increase in the overall incidence of diabetes since the 1960s into the 2000s [44]. Based on accounting data from the Associations of Statutory Health Insurance (SHI) Physicians, a decrease in the incidence of type 2 diabetes in women from 6.9 to 6.1 and in men from 8.4 to 7.7 per 1,000 persons was reported for 2014 through 2019, which was most evident in the older age groups [34] and was also observed in the present study. As the pandemic years were not included in the above-mentioned studies, it is not possible to compare the marked decrease in type 2 diabetes incidence in 2020 and the subsequent increase in 2021 observed in our study with these studies. Only one recent German study based on registry data of 6- to 18-year-olds includes data for the pandemic years and observed that the incidence of type 2 diabetes was higher in 2021, but not in 2020, than expected based on the trend in previous years (2011 to 2019) [45].

However, a decline in the utilisation of healthcare in the early phase of the pandemic was reported in many countries worldwide [46]. A decline in utilisation across all specialities was also noted in Germany during the first COVID-19 wave between March and mid-May 2020 [47–49]. The same was observed again, though to a lesser extent, during the second COVID-19 wave from October 2020 to mid-February 2021 [47, 48]. Between March and June 2021, the utilisation of medical services fluctuated and then, in the second half of 2021, exceeded pre-pandemic levels [47]. Against this background, it appears plausible that there was a decrease in the type 2 diabetes incidence in the first and second quarters of 2020 in the present study, in line with the decrease in utilisation observed in other studies. For persons with incident type 2 diabetes treated with medication, which presumably represented new cases with more severe symptoms, the observed decrease in incidence in the first year was less pronounced. Likewise, it seems reasonable that the subsequent increase in the overall incidence of type 2 diabetes observed in this study occurred almost parallel to the slow normalisation of utilisation. This might indicate a catch-up effect in the diagnosis of new cases. The result that the incidence of type 2 diabetes treated with medication was higher in all four quarters of 2021 than in previous years suggests that there might possibly be an additional effect of the pandemic beyond the catch-up effect of utilisation.

Thus, meta-analyses of studies comparing the incidence of diabetes in individuals with and without a previous COVID-19 disease suggest that SARS-CoV-2 infection may increase the risk of developing diabetes (by 62 % to 68 %) [29, 50] and type 2 diabetes (by 70 %) [29], respectively. In addition to inflammation-promoting processes, possible mechanisms include the binding of SARS-CoV-2 to specific receptors of metabolic tissues and organs such as pancreatic β-cells. The resulting deterioration in insulin secretion and action may lead to the development of diabetes or acceleration of the transition from a pre-diabetic phase to diabetes. However, the processes discussed are complex and not yet fully understood [29, 50].

### 4.3 Limitations

The present analysis of routine data for about nine million people with statutory health insurance in combination with data on GISD allows for a detailed calculation of the incidence of both type 1 and type 2 diabetes over time and differentiated by age, sex and regional socioeconomic deprivation. However, the use of routine data has its limitations.

The underlying InGef research database only includes data from statutory health-insured individuals of certain guild- and company-health insurance funds. A comparison with the population and hospital statistics from 2013 shows that the population of the InGef database was slightly younger on average and that the age-standardised morbidity was slightly lower compared to the overall German population [10]. According to an earlier study, the prevalence of diabetes of persons insured under different statutory health insurance schemes as well as of people insured under statutory and private health insurance schemes differs to some extent [51]. The standardisation used in the present study can compensate for differences in the age and sex distribution as compared to the population, but not with regard to other variables, so that a possible selection bias (‘health insurance bias’) remains. Moreover, the length of the diagnosis-free lead time can also have an impact on the estimated incidence [52]. Accordingly, a shorter diagnosis-free lead time can result in an overestimation of the incidence. Nevertheless, results on the incidence of type 2 diabetes from the years before the COVID-19 pandemic that are based on all individuals covered by statutory health insurance, with a diagnosis-free lead time of three years as a prerequisite [33, 34], are consistent with results on the corresponding years in the present study.

Furthermore, the data quality of the routine data depends on the documentation procedure. ICD-10 diagnoses from both the outpatient and inpatient settings were used to record new diabetes cases as validly and completely as possible, whereby a first outpatient diagnosis or secondary inpatient diagnosis had to be confirmed by another diagnosis in the following three quarters. Hereby, the exclusion of persons who died before confirmation of the diagnosis in a subsequent quarter can lead to an underestimation of the incidence. New cases that have not yet been diagnosed (e.g. undiagnosed diabetes) are not captured by routine data.

With regard to the differentiation of the incidence into type 1, type 2 and other forms of diabetes, misclassifications cannot be excluded in the present study. However, the differentiation was based on an established algorithm for differentiation of diabetes prevalence by type, which, in addition to the ICD-10 diagnosis codes also includes the type of anti-diabetic drugs prescribed, and, for ambiguous cases, further considers information on age and diagnostic pattern [13].

The results on the association between the incidence and regional socioeconomic deprivation are based on a linkage of data at the district level. Conclusions on the association of the individual socioeconomic situation with the development of diabetes can therefore not be drawn. Nevertheless, linking the routine data with the GISD provides valuable information that could not be derived from the used routine data alone.

### 4.4 Conclusion

Consistent with international data, the vast majority of new cases of diabetes in Germany is classified as type 2 diabetes, which develops particularly in mid- and later adulthood. Accordingly, a comparatively small proportion of new cases represents type 1 diabetes, often manifesting in childhood and adolescence. For both types of diabetes, there is an association with regional socioeconomic deprivation to the disadvantage of low deprived districts. In a global comparison, the incidence estimates of both types of diabetes in Germany are relatively high.

In line with possible indirect and direct COVID-19 effects under discussion, changes in the type-specific seasonal incidence patterns were observed in 2020 and 2021. For type 1 diabetes, these changes led to higher incidence estimates in 2020 and 2021 following after fluctuations in incidence between 2015 and 2019. For type 2 diabetes, after a declining trend, an incidence estimate comparable to 2015 was observed in 2021.

The results underline the need for further surveillance of the development of the incidence, for which a better availability of routine data of all statutory health insurance funds is desirable. In addition, the current increase in the incidence of both type 1 and type 2 diabetes in the context of the pandemic suggests that more attention is require for controlling the glucose metabolism levels in individuals with a history of SARS-CoV-2 infection. The relatively high incidence of type 1 diabetes as well as of type 2 diabetes in Germany points to the need for appropriate prevention strategies that also reduce the existing social inequalities in incidence.

## Key statement

- In total, 96.2 % of newly documented diabetes cases were classified as type 2 diabetes, 1.5 % as type 1 diabetes and 2.3 % as other forms of diabetes.
- Between 2015 and 2021, the incidence of type 1 diabetes increased from 9.5 to 11.6 new cases per 100,000 persons.
- Following an initial decrease, especially in the first year of the COVID-19 pandemic, the incidence of type 2 diabetes rose to 740 new cases per 100,000 persons in 2021.
- The specific seasonal patterns of the incidence of type 1 and type 2 diabetes observed in 2015 to 2019 were changed during the pandemic years of 2020 and 2021.
- The incidence of type 1 and type 2 diabetes was higher in regions with high socioeconomic deprivation than in regions with low deprivation.

## Figures and Tables

**Figure 4A:**
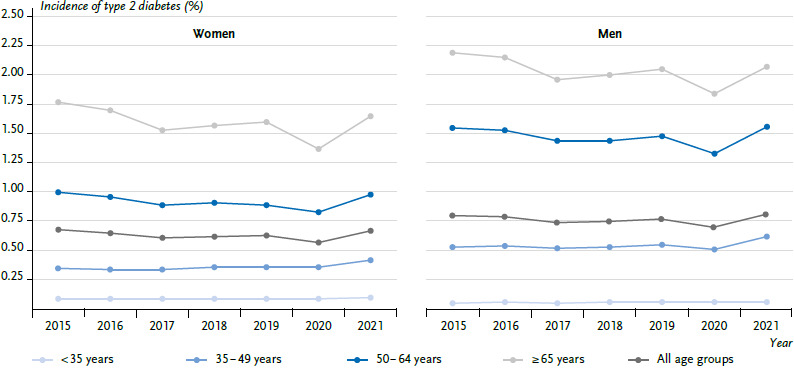
Temporal trend of the age-standardised incidence of type 2 diabetes in % with 95 % confidence interval by sex and age group (n per year: Annex Table 3) Source: InGef Research Database

**Figure 4B:**
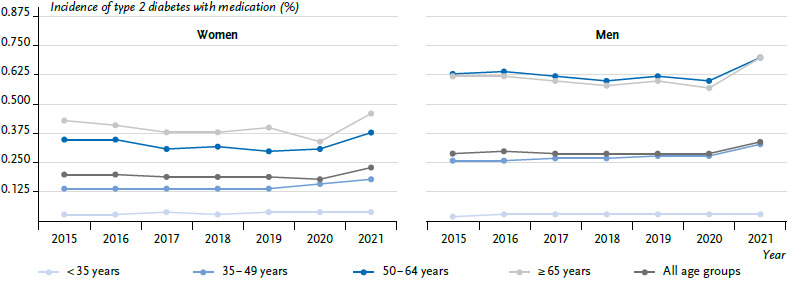
Temporal trend of the age-standardised incidence of type 2 diabetes with medication in % with 95 % confidence interval by sex and age group (n per year: Annex Table 3) Source: InGef Research Database

**Annex Table 1:** Criteria for the classification of the diabetes type for the estimation of the incidence of diabetes in routine data based on outpatient and inpatient diagnoses, medication prescriptions and age Source: own description

Type 1 Diabetes	Operationalisation
Case 1	[(E10 amb ≥ 2) OR (E10 stat ≥ 1)] AND (E11 amb = 0) AND (E11 stat = 0) AND (A10A ≥ 1) AND (A10B = 0)
Case 2	(E10 stat ≥ 1) AND (E11 amb = 0) AND (E11 stat = 0) AND (A10A = 0) AND (A10B = 0)
Case 3	(E10 amb = 1) AND (E11 amb = 0) AND (E11 stat = 0) AND (A10A ≥ 1) AND (A10B = 0)
Case 4	(E10 stat ≥ 1) AND (E11 amb ≥ 1) AND (E11 stat = 0) AND (A10A ≥ 1) AND (A10B = 0)
Case 5	(E10 amb ≥ 1) AND (E11 amb ≥ 1) AND (A10A ≥ 1) AND (A10B = 0) AND (AGE < 20 OR E10 amb > E11 amb^1^)
Case 6	(E10 stat ≥ 2) AND (E11 stat = 1) AND (A10A ≥ 1) AND (A10B = 0)
**Type 2 Diabetes**	**Operationalisation**
Case 1	[(E11 amb ≥ 2) OR (E11 stat ≥ 1)] AND (E10 amb = 0) AND (E10 stat = 0)
Case 2	[(E11 amb ≥ 1) OR (E11 stat ≥ 1)] AND (A10B ≥ 1)
Case 3	[(E11 amb ≥ 1) OR (E11 stat ≥ 1)] AND (E10 stat = 0) AND (A10A = 0)
Case 4	(E11 amb = 1) AND (E10 amb = 0) AND (E10 stat = 0) AND (E13 amb = 0) AND (E13 stat = 0) AND (A10A ≥ 1) AND (A10B = 0)
Case 5	(E11 amb ≥ 1) AND (E11 stat ≥ 1) AND (A10A = 0)
Case 6	[(E12 amb ≥ 1) OR (E14 amb ≥ 1)] AND (A10B ≥ 1)
Case 7	[(E12 amb ≥ 1) OR (E14 amb ≥ 1)] AND (E10 stat = 0) AND (A10A = 0) AND (A10B = 0)
Case 8	[(E11 amb ≥ 2) OR (E11 stat ≥ 1)] AND (E10 amb ≥ 1) AND (E10 stat = 0) AND (A10A = 0)
Case 9	[(E11 amb = 0) AND (E11 stat = 0) AND (E10 amb = 0) AND (E10 stat = 0) AND (E13 amb = 0) AND (E13 stat = 0)]
	AND [(E12 OR E14 amb ≥ 1) OR (E12 OR E14 stat ≥ 1)] AND (A10B ≥ 1)]
Case 10	(E11 amb ≥ 1) AND (E10 amb ≥ 1) AND (A10A ≥ 1) AND (A10B = 0) AND (AGE ≥ 20 AND E11 amb > E10 amb^2^)

amb: diagnosis confirmed in an outpatient setting or secondary inpatient diagnosis; stat: main inpatient diagnosis

^1^ Number of outpatient diagnoses E10 greater than number of outpatient diagnoses E11

^2^ Number of outpatient diagnoses E11 greater than number of outpatient diagnoses E10

**Annex Table 2:** Flowchart defining the study population, for each reporting year 2015 to 2021 Source: InGef Research Database

2015 N		2016 N	2017 N	2018 N	2019 N	2020 N	2021 N
Persons, insured for at least 1 day	6,782,717	6,915,843	6,947,163	7,001,805	7,060,976	7,064,881	7,121,060
Observable from 1.1. or birth until 31.12.	6,266,021	6,250,948	6,388,730	6,466,985	6,507,621	6,499,962	6,308,820
Observable in the previous year from 1.1. or birth to 31.12.	5,982,031	5,979,836	6,024,269	6,159,233	6,223,408	6,224,301	6,011,637
No outpatient or inpatient diabetes diagnosis and no prescription of an antidiabetic drug	5,465,174	5,454,276	5,490,349	5,616,459	5,671,939	5,668,763	5,478,769

**Annex Table 3:** Population at risk per reporting year stratified by sex and age groups Source: InGef Research Database

	2015		2016		2017		2018		2019		2020		2021	
	n	%	n	%	n	%	n	%	n	%	n	%	n	%
Women														
	**2,768,487**		**2,764,957**		**2,784,782**		**2,848,437**		**2,881,535**		**2,882,967**		**2,783,696**	
< 7 years	200,490	7.2 %	200,171	7.2 %	201,914	7.3 %	206,763	7.3 %	210,335	7.3 %	212,451	7.4 %	214,303	7.7 %
7 – 10 years	112,827	4.1 %	110,894	4.0 %	109,849	3.9 %	111,268	3.9 %	110,246	3.8 %	109,089	3.8 %	108,655	3.9 %
11 – 13 years	89,393	3.2 %	88,177	3.2 %	87,747	3.2 %	87,728	3.1 %	87,444	3.0 %	87,004	3.0 %	86,373	3.1 %
14 – 17 years	121,795	4.4 %	119,017	4.3 %	118,229	4.2 %	119,776	4.2 %	120,103	4.2 %	118,661	4.1 %	115,809	4.2 %
18 – 34 years	565,710	20.4 %	559,132	20.2 %	552,791	19.9 %	561,314	19.7 %	563,783	19.6 %	563,071	19.5 %	541,454	19.5 %
35 – 49 years	693,566	25.1 %	674,938	24.4 %	663,287	23.8 %	660,337	23.2 %	649,259	22.5 %	631,774	21.9 %	592,136	21.3 %
50 – 64 years	595,133	21.5 %	613,625	22.2 %	639,699	23.0 %	674,717	23.7 %	700,847	24.3 %	711,767	24.7 %	677,825	24.3 %
65 – 79 years	287,515	10.4 %	292,991	10.6 %	300,457	10.8 %	310,348	10.9 %	317,512	11.0 %	321,050	11.1 %	317,369	11.4 %
≥ 80 years	102,058	3.7 %	106,012	3.8 %	110,809	4.0 %	116,186	4.1 %	122,006	4.2 %	128,100	4.4 %	129,772	4.7 %
**Men**														
	**2,696,687**		**2,689,319**		**2,705,567**		**2,768,022**		**2,790,404**		**2,785,796**		**2,695,073**	
< 7 years	210,455	7.8 %	211,151	7.9 %	212,523	7.9%	217,720	7.9 %	221,276	7.9 %	223,541	8.0 %	225,629	8.4 %
7 – 10 years	118,612	4.4 %	116,189	4.3 %	115,064	4.3%	116,718	4.2 %	116,084	4.2 %	115,098	4.1 %	114,524	4.2 %
11 – 13 years	94,186	3.5 %	92,657	3.4 %	92,225	3.4%	92,325	3.3 %	91,714	3.3 %	91,048	3.3 %	90,330	3.4 %
14 – 17 years	127,324	4.7 %	124,781	4.6 %	123,947	4.6%	125,142	4.5 %	125,988	4.5 %	124,297	4.5 %	121,232	4.5 %
18 – 34 years	581,883	21.6 %	584,890	21.7 %	583,419	21.6 %	598,487	21.6 %	602,719	21.6 %	604,996	21.7 %	591,112	21.9 %
35 – 49 years	656,544	24.3 %	630,272	23.4 %	617,516	22.8%	614,948	22.2 %	600,788	21.5 %	585,328	21.0 %	555,799	20.6 %
50 – 64 years	576,675	21.4 %	593,900	22.1 %	617,984	22.8%	650,026	23.5 %	671,412	24.1 %	677,114	24.3 %	637,009	23.6 %
65 – 79 years	262,425	9.7 %	262,807	9.8 %	265,737	9.8 %	270,642	9.8 %	273,066	9.8 %	271,795	9.8 %	265,590	9.9 %
≥ 80 years	68,583	2.5 %	72,672	2.7 %	77,152	2.9 %	82,014	3.0 %	87,357	3.1 %	92,579	3.3 %	93,848	3.5 %

**Annex Table 4:** Age-standardised incidence of type 1 and type 2 diabetes over time (2015 – 2021) stratified by sex and age (n per year: Annex Table 3) Source: InGef Research Database

Type 1 diabetes	2015		2016		2017		2018		2019		2020		2021	
	per	(95 % CI)	per	(95 % CI)	per	(95 % CI)	per	(95 % CI)	per	(95 % CI)	per	(95 % CI)	per	(95 % CI)
	100,000		100,000		100,000		100,000		100,000		100,000		100,000	
Women														
< 7 years	21.2	(19.6 – 23.0)	24.4	(22.6 – 26.3)	18.5	(16.9 – 20.2)	21.3	(19.7 – 23.1)	22.5	(20.8 – 24.4)	21.4	(19.8 – 23.3)	25.3	(23.5 – 27.3)
7 – 10 years	32.9	(30.1 – 36.0)	31.4	(28.7 – 34.4)	27.3	(24.7 – 30.1)	36.4	(33.5 – 39.6)	30.6	(27.9 – 33.6)	34.9	(32.0 – 38.0)	44.1	(40.9 – 47.7)
11 – 13 years	33.6	(30.3 – 37.2)	17.0	(14.7 – 19.6)	23.8	(21.1 – 26.9)	14.8	(12.6 – 17.2)	35.4	(32.0 – 39.1)	55.2	(50.9 – 59.8)	39.9	(36.3 – 43.8)
14 – 17 years	17.2	(15.2 – 19.5)	14.2	(12.4 – 16.3)	22.7	(20.4 – 25.3)	22.5	(20.2 – 25.0)	14.2	(12.4 – 16.3)	19.4	(17.2 – 21.8)	20.7	(18.5 – 23.1)
18 – 34 years	9.7	(9.1 – 10.5)	10.6	(9.9 – 11.3)	9.1	(8.4 – 9.8)	11.9	(11.2 – 12.7)	9.7	(9.1 – 10.5)	11.4	(10.6 – 12.1)	11.9	(11.2 – 12.7)
35 – 49 years	4.8	(4.3 – 5.3)	5.0	(4.5 – 5.5)	3.0	(2.7 – 3.5)	6.5	(5.9 – 7.1)	5.7	(5.2 – 6.3)	6.4	(5.8 – 7.0)	4.4	(4.0 – 4.9)
≥ 50 years	3.1	(2.9 – 3.4)	2.9	(2.7 – 3.2)	3.5	(3.2 – 3.8)	3.2	(2.9 – 3.5)	2.4	(2.2 – 2.7)	2.4	(2.1 – 2.6)	2.8	(2.5 – 3.1)
**All ages**	**8.7**	**(8.4 – 9.0)**	**8.3**	**(8.1 – 8.6)**	**7.8**	**(7.6 – 8.1)**	**9.2**	**(8.9 – 9.5)**	**8.5**	**(8.2 – 8.7)**	**9.7**	**(9.4 – 10.0)**	**9.9**	**(9.6 – 10.2)**
**Men**														
< 7 years	17.2	(15.7 – 18.7)	20.4	(18.8 – 22.1)	20.1	(18.6 – 21.8)	21.9	(20.3 – 23.7)	19.5	(17.9 – 21.1)	23.1	(21.4 – 24.9)	33.6	(31.6 – 35.8)
7 – 10 years	40.5	(37.4 – 43.8)	40.2	(37.1 – 43.4)	29.6	(27.0 – 32.4)	32.6	(29.9 – 35.6)	45.5	(42.3 – 49.0)	43.4	(40.2 – 46.8)	37.4	(34.5 – 40.6)
11 – 13 years	36.1	(32.8 – 39.8)	29.1	(26.2 – 32.4)	35.9	(32.6 – 39.5)	41.9	(38.4 – 45.9)	30.5	(27.5 – 33.9)	58.4	(54.2 – 63.0)	58.6	(54.3 – 63.1)
14 – 17 years	22.8	(20.5 – 25.3)	35.1	(32.3 – 38.2)	31.5	(28.9 – 34.5)	27.1	(24.6 – 29.8)	24.5	(22.1 – 27.1)	34.5	(31.7 – 37.5)	23.2	(20.9 – 25.7)
18 – 34 years	11.8	(11.1 – 12.6)	13.1	(12.4 – 13.9)	15.1	(14.3 – 15.9)	12.7	(11.9 – 13.5)	15.0	(14.2 – 15.8)	14.0	(13.3 – 14.8)	14.1	(13.3 – 14.9)
35 – 49 years	8.0	(7.3 – 8.6)	7.0	(6.4 – 7.6)	6.5	(5.9 – 7.1)	6.9	(6.3 – 7.5)	8.3	(7.7 – 9.0)	10.8	(10.1 – 11.6)	8.6	(7.9 – 9.3)
≥ 50 years	2.4	(2.2 – 2.7)	3.7	(3.4 – 4.0)	4.0	(3.7 – 4.4)	3.9	(3.6 – 4.3)	4.6	(4.2 – 5.0)	3.5	(3.2 – 3.8)	3.6	(3.3 – 3.9)
**All ages**	**10.4**	**(10.1 – 10.7)**	**11.5**	**(11.2 – 11.9)**	**11.6**	**(11.2 – 11.9)**	**11.3**	**(11.0 – 11.7)**	**12.3**	**(11.9 – 12.7)**	**13.7**	**(13.3 – 14.0)**	**13.3**	**(13.0 – 13.7)**
**Type 2 diabetes**	**2015**		**2016**		**2017**		**2018**		**2019**		**2020**		**2021**	
	**%**	**(95 % CI)**	**%**	**(95 % CI)**	**%**	**(95 % CI)**	**%**	**(95 % CI)**	**%**	**(95 % CI)**	**%**	**(95 % CI)**	**%**	**(95 % CI)**
**Women**														
< 18 years	0.017	(0.016 – 0.018)	0.015	(0.014 – 0.016)	0.016	(0.015 – 0.017)	0.014	(0.013 – 0.014)	0.015	(0.015 – 0.016)	0.014	(0.013 – 0.015)	0.014	(0.013 – 0.015)
18 – 34 years	0.15	(0.15 – 0.16)	0.16	(0.16 – 0.16)	0.16	(0.15 – 0.16)	0.15	(0.15 – 0.16)	0.16	(0.15 – 0.16)	0.16	(0.15 – 0.16)	0.17	(0.16 – 0.17)
35 – 49 years	0.35	(0.35 – 0.35)	0.34	(0.33 – 0.34)	0.34	(0.34 – 0.34)	0.36	(0.35 – 0.36)	0.36	(0.36 – 0.37)	0.36	(0.36 – 0.36)	0.42	(0.42 – 0.43)
50 – 64 years	1.00	(1.00 – 1.01)	0.96	(0.95 – 0.96)	0.89	(0.89 – 0.90)	0.91	(0.91 – 0.92)	0.89	(0.89 – 0.90)	0.83	(0.82 – 0.84)	0.98	(0.97 – 0.98)
65 – 79 years	1.76	(1.75 – 1.77)	1.69	(1.68 – 1.70)	1.50	(1.49 – 1.51)	1.57	(1.56 – 1.59)	1.60	(1.59 – 1.61)	1.39	(1.38 – 1.40)	1.66	(1.65 – 1.68)
≥ 80 years	1.80	(1.78 – 1.81)	1.71	(1.69 – 1.73)	1.58	(1.56 – 1.59)	1.56	(1.54 – 1.57)	1.60	(1.59 – 1.62)	1.33	(1.32 – 1.35)	1.62	(1.60 – 1.63)
**All ages**	**0.68**	**(0.68 – 0.68)**	**0.65**	**(0.65 – 0.65)**	**0.61**	**(0.60 – 0.61)**	**0.62**	**(0.62 – 0.62)**	**0.63**	**(0.62 – 0.63)**	**0.57**	**(0.56 – 0.57)**	**0.67**	**(0.67 – 0.67)**
**Men**														
< 18 years	0.011	(0.011 – 0.012)	0.009	(0.009 – 0.010)	0.012	(0.011 – 0.013)	0.013	(0.013 – 0.014)	0.012	(0.011 – 0.013)	0.010	(0.009 – 0.011)	0.013	(0.012 – 0.014)
18 – 34 years	0.09	(0.08 – 0.09)	0.09	(0.09 – 0.10)	0.09	(0.09 – 0.09)	0.09	(0.09 – 0.09)	0.10	(0.10 – 0.10)	0.09	(0.09 – 0.10)	0.11	(0.10 – 0.11)
35 – 49 years	0.53	(0.52 – 0.53)	0.54	(0.53 – 0.54)	0.52	(0.52 – 0.53)	0.53	(0.52 – 0.53)	0.55	(0.54 – 0.55)	0.51	(0.51 – 0.52)	0.62	(0.61 – 0.62)
50 – 64 years	1.55	(1.54 – 1.56)	1.53	(1.53 – 1.54)	1.44	(1.44 – 1.45)	1.44	(1.43 – 1.45)	1.48	(1.47 – 1.49)	1.33	(1.32 – 1.34)	1.56	(1.56 – 1.57)
65 – 79 years	2.21	(2.20 – 2.22)	2.16	(2.14 – 2.17)	2.01	(2.00 – 2.03)	2.07	(2.06 – 2.08)	2.12	(2.11 – 2.14)	1.90	(1.88 – 1.91)	2.17	(2.15 – 2.18)
≥ 80 years	2.13	(2.11 – 2.16)	2.12	(2.10 – 2.15)	1.84	(1.82 – 1.86)	1.81	(1.79 – 1.84)	1.86	(1.84 – 1.88)	1.69	(1.67 – 1.71)	1.83	(1.81 – 1.86)
**All ages**	**0.80**	**(0.80 – 0.80)**	**0.79**	**(0.79 – 0.79)**	**0.74**	**(0.74 – 0.74)**	**0.75**	**(0.75 – 0.75)**	**0.77**	**(0.77 – 0.77)**	**0.70**	**(0.70 – 0.70)**	**0.81**	**(0.81 – 0.81)**

**Annex Table 5:** Age-standardised incidence of type 1 and type 2 diabetes stratified by regional socioeconomic deprivation, sex and reporting year (n per year: Annex Table 3) Source: InGef Research Database; GISD Release 2022 v0.1

	Female						Male						Total					
	2019		2020		2021		2019		2020		2021		2019		2020		2021	
Type 1 diabetes (New cases per 100,000 (95 % CI))																		
**< 18 years**																		
Low	25.7	(23.6 – 28.1)	25.8	(23.6 – 28.1)	28.5	(26.2 – 30.9)	27.4	(25.3 – 29.8)	32.4	(30.1 – 35.0)	36.5	(34.0 – 39.2)	26.6	(25.1 – 28.3)	29.2	(27.6 – 30.9)	32.6	(30.9 – 34.4)
Medium	20.5	(19.1 – 21.9)	27.2	(25.7 – 28.9)	27.7	(26.1 – 29.4)	26.5	(25.0 – 28.1)	34.7	(33.0 – 36.5)	32.4	(30.8 – 34.2)	23.6	(22.6 – 24.7)	31.1	(29.9 – 32.3)	30.2	(29.0 – 31.4)
High	43.0	(38.6 – 47.8)	50.1	(45.4 – 55.3)	52.5	(47.7 – 57.8)	36.2	(32.3 – 40.5)	49.5	(45.0 – 54.5)	53.6	(48.9 – 58.8)	39.5	(36.6 – 42.7)	49.8	(46.5 – 53.3)	53.1	(49.7 – 56.8)
≥ **18 years**																		
Low	4.1	(3.7 – 4.5)	4.7	(4.2 – 5.1)	6.4	(5.9 – 7.0)	8.2	(7.6 – 8.9)	7.1	(6.6 – 7.8)	6.7	(6.2 – 7.3)	6.1	(5.7 – 6.5)	5.9	(5.5 – 6.2)	6.6	(6.2 – 7.0)
Medium	5.5	(5.2 – 5.9)	5.7	(5.4 – 6.1)	5.2	(4.9 – 5.6)	8.4	(7.9 – 8.8)	8.8	(8.4 – 9.3)	7.9	(7.5 – 8.4)	6.9	(6.6 – 7.2)	7.2	(7.0 – 7.5)	6.5	(6.3 – 6.8)
High	4.5	(3.9 – 5.2)	6.6	(5.9 – 7.4)	4.5	(3.9 – 5.2)	10.2	(9.2 – 11.2)	8.5	(7.6 – 9.5)	10.1	(9.2 – 11.2)	7.3	(6.7 – 7.9)	7.5	(7.0 – 8.2)	7.3	(6.7 – 7.9)
**Type 2 diabetes (% (95 % CI))**																		
Low	0.58	(0.58 – 0.58)	0.53	(0.53 – 0.53)	0.61	(0.61 – 0.61)	0.71	(0.71 – 0.71)	0.64	(0.64 – 0.65)	0.73	(0.73 – 0.73)	0.65	(0.65 – 0.65)	0.59	(0.59 – 0.59)	0.67	(0.67 – 0.67)
Medium	0.64	(0.64 – 0.64)	0.57	(0.57 – 0.57)	0.67	(0.67 – 0.67)	0.78	(0.78 – 0.78)	0.71	(0.71 – 0.71)	0.83	(0.83 – 0.83)	0.71	(0.71 – 0.71)	0.64	(0.64 – 0.64)	0.75	(0.75 – 0.75)
High	0.71	(0.71 – 0.71)	0.65	(0.65 – 0.65)	0.82	(0.82 – 0.82)	0.90	(0.90 – 0.90)	0.79	(0.79 – 0.79)	0.93	(0.93 – 0.93)	0.80	(0.80 – 0.80)	0.72	(0.72 – 0.72)	0.87	(0.87 – 0.87)

low = 20 % of the districts with the lowest deprivation, medium = 60 % of the districts with medium deprivation, high = 20 % of the districts with the highest deprivation
